# Transcriptional Regulatory Networks Associate with Early Stages of Potato Virus X Infection of *Solanum tuberosum*

**DOI:** 10.3390/ijms22062837

**Published:** 2021-03-11

**Authors:** Venura Herath, Jeanmarie Verchot

**Affiliations:** 1 Department of Plant Pathology and Microbiology, Texas A&M University, College Station, TX 77802, USA; venura@agri.pdn.ac.lk; 2 Department of Agriculture Biology, Faculty of Agriculture, University of Peradeniya, Peradeniya 20400, Sri Lanka

**Keywords:** PVX, gene expression regulation, RNA-seq, *Solanum tuberosum*, potato virology, virus movement, host-pathogen interactions, antiviral defense, potexvirus

## Abstract

*Potato virus X* (PVX) belongs to genus *Potexvirus.* This study characterizes the cellular transcriptome responses to PVX infection in Russet potato at 2 and 3 days post infection (dpi). Among the 1242 differentially expressed genes (DEGs), 268 genes were upregulated, and 37 genes were downregulated at 2 dpi while 677 genes were upregulated, and 265 genes were downregulated at 3 dpi. DEGs related to signal transduction, stress response, and redox processes. Key stress related transcription factors were identified. Twenty-five pathogen resistance gene analogs linked to effector triggered immunity or pathogen-associated molecular pattern (PAMP)-triggered immunity were identified. Comparative analysis with Arabidopsis unfolded protein response (UPR) induced DEGs revealed genes associated with UPR and plasmodesmata transport that are likely needed to establish infection. In conclusion, this study provides an insight on major transcriptional regulatory networked involved in early response to PVX infection and establishment.

## 1. Introduction

*Potato virus X* (PVX) is a positive-strand RNA virus that infects potatoes, although primarily studied in model hosts, *Nicotiana benthamiana* and Arabidopsis. In *N. benthamiana* leaves inoculated with the PVX infectious clone containing the green fluorescent protein (GFP) gene [1,2,3], the primary infection occurs in foci that expand radially by cell-to-cell movement in all directions. PVX-GFP fluorescent foci typically appear at a macroscopic level within the first 2 or 3 days post-inoculation (dpi). Around 5 dpi, the virus typically reaches nearby veins and begins to load into the phloem for transport out of the inoculated leaf to unload in the upper leaves [4,5,6,7]. The time-frame for virus entry into the vascular system is contingent upon the density of vascular patterning and leaf dimensions [8,9]. After PVX-GFP enters the phloem, it is transported to multiple distal regions of the plant to establish systemic infection.

Within the first few hours or days of infection, viruses enact critical steps to establish themselves in the host cell and initiate replication. PVX engages chaperones and other proteins into replication complexes [10,11,12] and alters cellular membranes to build scaffolds. PVX also affects the cell’s program to protect against host defenses and suppresses post-transcriptional gene silencing [13]. Recent evidence suggests that virus effector-proteins target regulatory nodes of cellular pathways to create an environment that favors infection [14,15]. For example, viral silencing suppressor proteins interfere with different steps of the RNAi machinery [16]. Small membrane-binding proteins embedded in the endoplasmic reticulum manipulate the unfolded protein response machinery [17]. Other targets include autophagy, RNA granules, vesicle trafficking, lipid metabolism, and phytohormones [18]. Moreover, some cellular pathways are not globally conserved, such as those between monocot and dicots, or the defense-related steroidal glycoalkaloids (SGAs) in *Solanum* species [19,20]. Therefore, it is necessary to investigate the cellular responses to virus infection in agronomic hosts if we ever expect to use science to deploy sensible crop improvement strategies.

During the past decade, most “omic” approaches to uncover the transcriptional and metabolic responses to plant virus infection used systemically infected model hosts, although a few studies used microarray or RNA-seq technologies to report transcription, small interfering RNA, or micro RNA profiles in potato during infection with potato virus Y, or potato spindle tuber viroid [21,22,23,24]. Few studies investigate the early responses to virus infection model or agronomic hosts, and there are no studies until now using PVX in potato. This study was undertaken to investigate the cellular pathways in *S. tuberosum* that are potential targets for PVX exploitation during the radial expansion of primary infection foci. For this, we analyzed gene expression changes at 2 and 3 dpi with PVX by RNA-Seq. This next-generation technology took advantage of the recently updated version 6.1 genome sequence of DM1-3 516 R44, a doubled monoploid clone of *S. tuberosum*, representing a high-quality annotation dataset [25]. The data presented in this study identified a small set of genes that likely play important roles in establishing virus infection.

## 2. Results

### 2.1. Inoculation of Potato Plants with PVX-GFP and Evaluation of Virus Infection

Potato plants were inoculated with plant sap containing PVX-GFP virions by rub inoculation prior to RNAseq analysis. To standardize the virus inoculum for application to the *S. tuberosum* (cv Russet Norkotah) leaves, the Holmes’ assay was carried out using *Chenopodium quinoa* plants, a known local lesion host for PVX. The Holme’s assay is based on the premise that the virus titer is proportional to the dilution of the extracts that can be visualized and counted using a local lesion host. This Holmes’ local lesion assay is a standard approach to reporting the proportion of virus in a volume of extract and for comparing infectivity of different inocula sources [26]. *C. quinoa* and potato plants were maintained in a growth room at a constant temperature of 23 °C and 16 h light. A dilution series of soluble leaf homogenate was prepared from *N. benthamiana* plant that was systemically infected with PVX-GFP and then rub-inoculated onto *Chenopodium quinoa* leaves. Lesions first appear as chlorotic spots and expand to necrotic sports surrounded by green rings (Figure S1A,B). We selected an inoculum of 20 µL of a 1:5 (*w*/*v*) dilution in phosphate buffer that produced an average of 45 foci per leaf (*n* = 5) [27] to use in subsequent potato experiments (Figure S1C). Next, this inoculum was applied to potato leaves and then PVX-GFP fluorescence was viewed using a stereo fluorescent microscope and 20× objective. At 2 and 3 dpi and there was an average of 27 infection foci per inoculated leaf (*n* = 4; Figure S1D,E). Using the Holme’s assay combined with viewing GFP was important to monitor the titer of PVX-GFP inoculum and distribution of infection foci across leaves for determining the sampling time. This was needed to satisfy a model of virus fitness proposed by Rodrigo et al. (2014) which implies that the appropriate abundance of primary infection foci during this period of 2–3 days represents conditions that support robust replication and cell-to-cell movement toward establishing systemic infection [28]. Infected leaves were harvested and RNA was extracted. PVX-GFP infection was further confirmed RT-PCR.

### 2.2. A Genome-Wide Analysis of Differentially Expressed Genes (DEGs) in Potato Inoculated with PVX-GFP

An RNA-seq study was conducted using the BGISEQ-500 platform to capture the transcriptome changes occurring during the early stages of infection which likely occur to support virus replication and cell-to-cell movement. We inoculated nine single largest leaflets of compound potato leaves. Three leaflets were pooled and RNA was extracted at 2 and 3 dpi. Transcription profiles were determined for three replicate samples at 2 and 3 dpi. We obtained 453.39 million raw and 406.91 million clean read pairs mapping to the reference genome using HISAT2 software (Table S1). A total of 336.97 million read pairs were uniquely mapped to the reference genome representing approximately 83% of the clean reads. The total alignment rate including unique and multi-mapped reads with the reference genome was 89.75% indicating this is a quality dataset.

**Figure 1:**
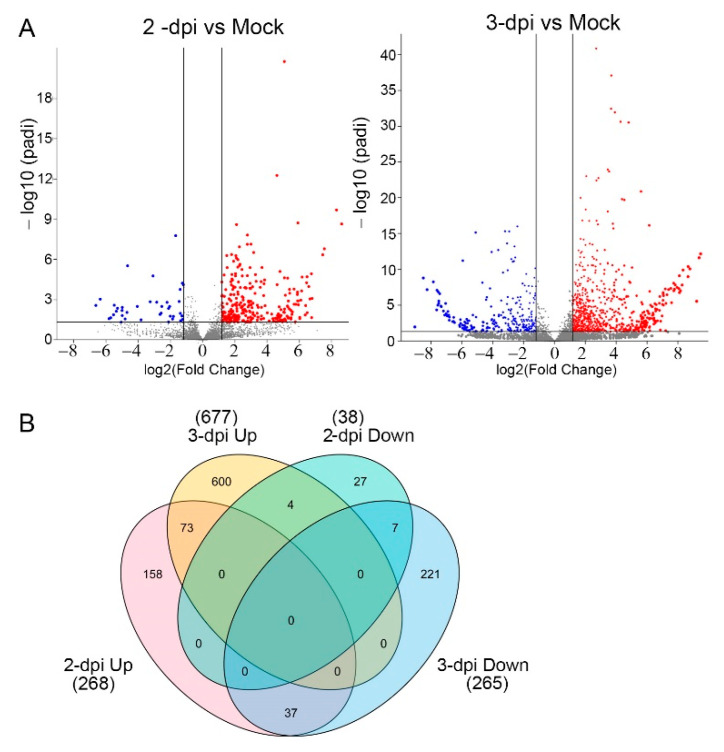
Differentially expressed number of genes (DEGs). (**A**) Volcano plots show the significantly upregulated genes in blue and downregulated genes in red (*p* < 0.05). (**B**) Venn diagram highlighting unique and common DEGs in PVX-GFP infected leaves at 2 and 3 dpi across multiple comparisons using a −log10 adjusted *p*-value.

The total number of differentially expressed genes (DEGs) are represented in volcano plots with the upregulated transcripts identified in red and, the downregulated transcripts in blue (Figure 1A). A Venn diagram of transcripts (Figure 1B) identifies a total of 306 DEGs at 2 dpi, which is the sum of 268 upregulated and 37 downregulated genes which are listed in Tables S2 and S3. At 3dpi, there were 942 DEGs, including 677 upregulated and 265 downregulated genes. At 2 and 3 dpi, 73 similar genes were upregulated, and seven similar genes were downregulated. Four genes were downregulated at 2 dpi but upregulated at 3 dpi. Thirty-seven genes were upregulated at 2 dpi but downregulated at 3 dpi (Figure 1B, Tables S2 and S3).

**Figure 2:**
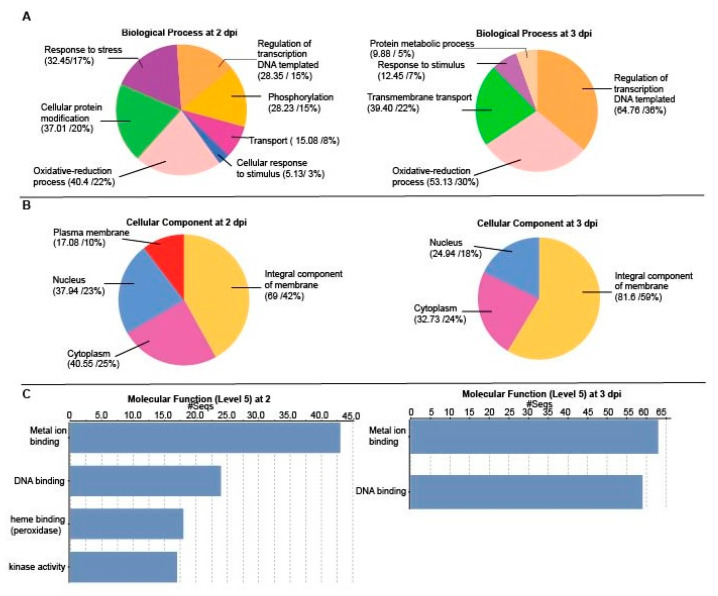
Summary pie charts and bar charts of GO analysis of DEGs that are upregulated transcripts at 2 and 3 dpi. The charts show the distribution of sequences according to the level 5 GO terms affiliated with (**A**) biological processes, (**B**) cellular components, and (**C**) molecular functions. The numbers and percentages of sequences are presented in parentheses with each slice in the pie charts.

Gene Ontology (GO) describes gene products in the categories of biological process, molecular function, and cellular component. To understand the complex expression patterns portrayed in Figure 1B, we performed GO enrichment analysis to cluster proteins of similar functions within the DEG datasets (Figure 2) [29]. Regarding the upregulated genes, we found a GO IDs associating with biological processes at 2 dpi included oxidative stress processes which made up 22% and cellular protein modification which was 20% of sequences (Figure 2A). Two similar terms which might be considered together are “response to stress” and “cellular response to stimuli” which corresponded to 17% and 3% of gene sequences. Response to transcription and phosphorylation were each 15% and the transport was 8%. At 3 dpi, regulation of transcription and oxidative stress each comprised 30% of sequences. Transmembrane transport was 22% and protein metabolic processes represented 5% of sequences (Figure 2A). Regarding cellular component, integral membrane proteins comprised 42% of sequences at 2 dpi and 59% at 3 dpi. Cytoplasmic factors represented 25% and 24% of sequences at 2 and 3 dpi, respectively (Figure 2B). Genes associated with the nucleus representing 23% of sequences at 2 dpi and 18% of sequences at 3 dpi. Genes associated with the plasma membrane represented 10% of sequences at 2 dpi (Figure 2B). At 2 dpi, four GO terms for molecular functions were identified: metal ion binding, DNA binding, heme-binding/peroxidase activity, and kinase activity (Figure 2C). At 3 dpi, there are two GO categories of molecular functions: metal ion binding and DNA binding. Metal ion binding points to potential enzymatic activities, kinase activities suggest signal transduction cascades, and DNA binding points to transcriptional regulation.

**Figure 3:**
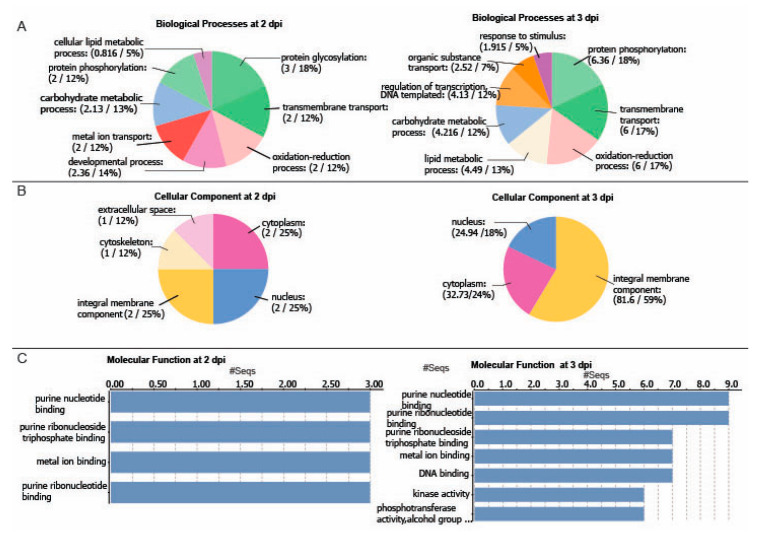
Summary pie charts and bar charts of GO analysis of DEGs that are downregulated transcripts at 2 and 3 dpi. The charts show the distribution of sequences according to the GO terms affiliated with (**A**) biological processes, (**B**) cellular components, and (**C**) molecular functions. The numbers and percentages of sequences are presented in parentheses with each slice in the pie charts.

Next, we searched the GO terms for downregulated genes at 2 and 3 dpi (Figure 3A). At 2 dpi the biological processes were distributed between 12% and 18% of total sequences associating with protein glycosylation and phosphorylation, transmembrane transport, oxidative-reduction process, developmental processes, metal ion transport, and carbohydrate metabolic process. Cellular lipid metabolic processes were 5% of sequences. At 3 dpi the sequences were distributed from 5% to 18% of the total across protein phosphorylation, transmembrane transport, oxidation-reduction process, lipid metabolic process, carbohydrate metabolic process, regulation of transcription, organic substance transport, and response to stimuli (Figure 3A). Noting that similar GO IDs occur in upregulated and downregulated datasets suggest that PVX might target and carefully manipulate specific pathway regulatory nodes.

Examining the cellular components GO terms, at 2 dpi between 12 and 25% of total sequences associate with the cytoplasm, nucleus, integral membrane component, cytoskeleton and extracellular space (Figure 3B). At 3 dpi there were only three cellular components GO terms with 18% of sequences representing the nucleus, 24% associating with the cytoplasm, and 59% associating with integral membrane component (Figure 3B). Four downregulated molecular functions at 2 dpi (Figure 3C) were: purine nucleotide binding, purine ribonucleoside triphosphate binding, purine ribonucleotide binding and, metal ion binding. At 3 dpi, the categories of molecular functions expand to include DNA binding, kinase activity, and phosphotransferase activity.

### 2.3. Differentially Regulated Transcription Factors (TFs) at 2 and 3 dpi

**Figure 4:**
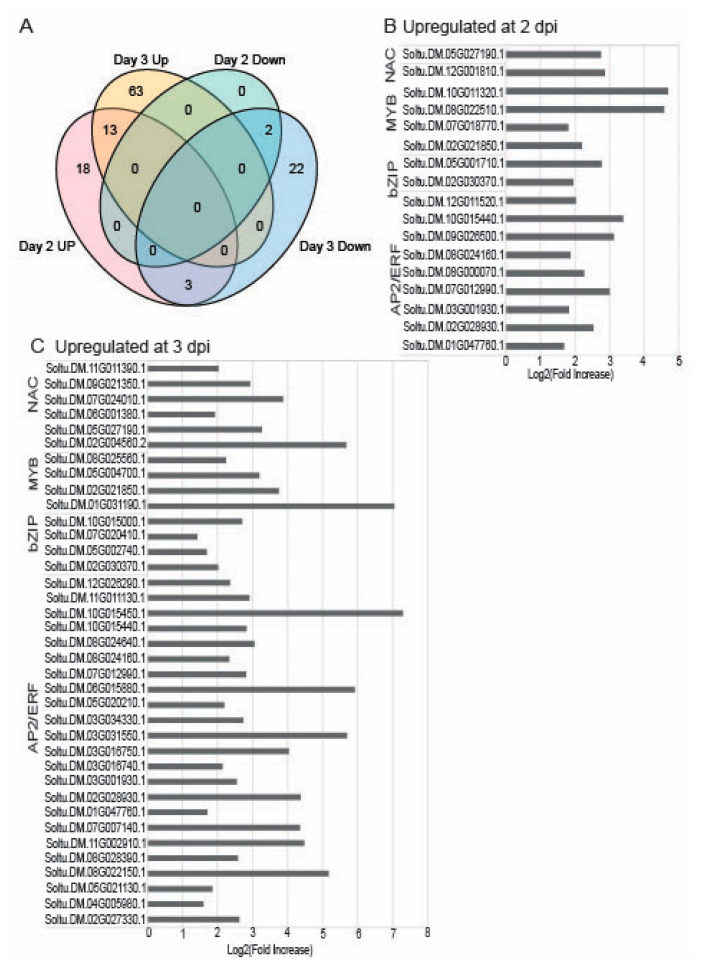
Differentially expressed transcription factors (TFs). (**A**) Venn diagram visualizing the overlap of differentially expressed TFs at 2 and 3 dpi. (**B**,**C**) Bar graphs show identifiers for genes that are upregulated at 2 and 3 dpi, respectively, with a cut-off value of >1.0. The TF families include: NAC, MYB, bZIP, and AP2/ERF.

TFs within the AP2/ERF, bZIP, MYB, NAC, and WRKY families control important nodes in gene regulatory networks and metabolic adjustment to environmental factors [30,31]. Investigating TFs that target genes in abiotic and biotic stress tolerance is essential toward understanding traits at the molecular level that may be useful for genetic selection in breeding or engineering stress-tolerant crops [32,33]. We searched the PVX-induced potato transcriptome for TFs and then identified the Arabidopsis orthologues. The Venn diagram in Figure 4A shows that 31 TFs were upregulated at 2 dpi and 63 TFs at 3 dpi with thirteen TFs commonly upregulated at both days (Figure 4A and Table S4). There were two TFs downregulated at 2 dpi contributing to 24 TFs downregulated at 3 dpi (Figure 4A, Table S5). We tabulated factors belonging to 24 TF families that are upregulated and 13 TF families that are downregulated (Figure 4, Tables S4 and S5).

We focused our attention on four TFs families that are known to contribute to environmental stress responses in plants NAC, MYB, bZIP, and AP2/ERF noting that there was between 1.5- to 8-fold increase in gene expression due to PVX-GFP infection (Figure 4B,C). Combining 2 and 3 dpi datasets, thirty-three upregulated TFs belong to the APATELA2 (AP2) family (Table S4) which is involved in responses to abiotic stress and phytohormone signaling. The AP2/ethylene-responsive factor (AP2/ERF) family is crucial for regulating a network of genes that promote plant survival to environmental stresses [30,31,34]. The dehydration-responsive element binding (DREB) factors are a subfamily of AP2/ERF TFs [35]. Interestingly, the potato homologs for the Arabidopsis C-repeat binding factor 2 (CBF2)/DREB1C, CBF4/DREB1D, DREB26 were upregulated which also engage in drought and cold tolerance (Table S4) [35].

The bZIP TF family is central to the regulation of developmental and physiological responses as well as abiotic and biotic stress responses [36,37,38]. Five of TFs (*StbZIP37*, *StbZIP42*, *StbZIP46*, *StbZIP58*, and *StbZIP61*) are upregulated early in PVX infection and only one (*StbZIP72*) is downregulated (Tables S4 and S5). Prior studies showed *StbZIP60* is induced at 5 dpi following PVX infection in potatoes and its absence from this dataset suggests it is not among the earliest TFs that are induced by PVX infection [15,17].

In plants, MYB TFs are involved in responses to biotic and abiotic stress, development, metabolism, cell cycle control, and defense [39,40,41,42]. Nine MYB factors are upregulated and eight are downregulated at 2 or 3 dpi (Tables S4 and S5). The Arabidopsis orthologs (*MYB3*, *MYB15*, *MBY61*, *MYB85*, and *MYB125/DUO1*) are involved in ABA responses, cold tolerance, secondary metabolism, stomata aperture, lignin biosynthesis, and pollen development [43]. Interestingly, the downregulated *MYB83* is a known target for the microRNA (miRNA)159c that is also activated by drought stress in Arabidopsis [43].

The NAC factors are plant-specific TFs and are involved in embryonic, floral, and vegetative development. NAC factors are also involved in lateral root formation, auxin signaling, pathogen defense, abiotic stress, and ER stress. Early research identified a *StNAC* factor involved in responses to *Phytophthora infestans* [44]. AtNAC28 binds to the turnip crinkle virus (TCV) capsid leading to a hypersensitive response. Looking at the dataset in Table S4, the potato homolog of *AtNAC28* is also upregulated as well as six other NACs that have not yet been characterized [44,45].

The WRKY TF family in plants mediate responses to pathogens infection, wounding, drought, and cold. On the potato ortholog for *WRKY41* is upregulated at 2 dpi and then down-regulated at 3 dpi.

### 2.4. Identification of Upregulated Pathogen Resistance Gene Analogs (RGA)

**Figure 5:**
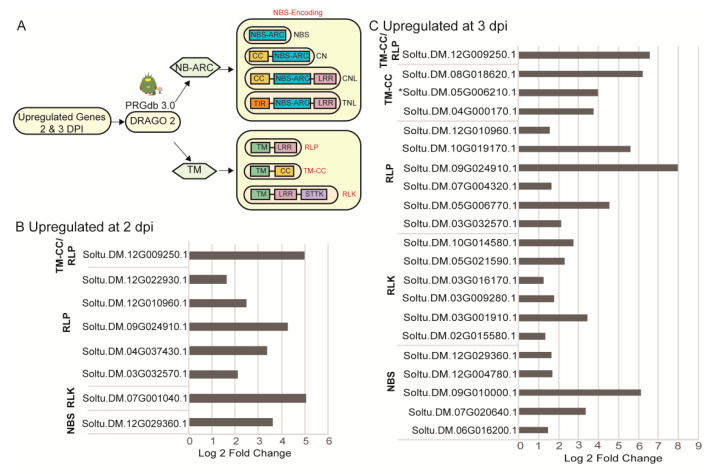
Upregulated RGAs identified using the DRAGO 2 pipeline. (**A**) Diagrammatic representation of the discovery pipeline based on protein domains such as nucleotide-binding site (NB-ARC), leucine-rich repeat (LRR), transmembrane domain (TM), coiled-coil (CC), Toll/Interleukin-1 receptor (TIR), lysin motif (LysM), and the serine/threonine and tyrosine kinase (STTK). RGA categories are identified in red: NBS-encoding, RLP, TM-CC, or RLK. Subcategories within the NBS-encoding genes are identified inside yellow bubbles. (**B**,**C**) Bar graphs show genes that were upregulated at 2 and 3 dpi, respectively. The putative gene families are identified on the left. Asterisk places next to Sotu.DM.05G006210 identifies a gene that is classified at TM-CC but also has an NB-ARC domain so it potentially can be classified as a CNL.

Considering the identified GO terms for biological processes included responses to stimuli and oxidative reduction, alongside kinase activities as a prominent GO term for molecular functions, we hypothesized that pathogen innate or adaptive immune systems are likely engaged in early responses to infection [46]. R genes linked to host immunity typically have transmembrane (TM) domains, nucleotide-binding site (NBS) and leucine-rich repeats (LRR) [47,48,49,50] and activate signaling cascades to alter nuclear gene expression. Salicylic acid activates mitochondrial signaling and affects redox regulation in a manner that is known to inhibit virus replication and movement [46,49,50]. While most R genes are transcribed in tissues at basal levels, there are examples where pathogen challenge induces R gene expression [51,52,53,54]. Therefore, we used the Disease Resistance Analysis and Gene Orthology (DRAGO 2) pipeline of RGAugury to predict RGAs in the PVX induced transcriptome (Figure 5A) [55,56].

The CNL (CC-NBS-LRR) and TNL (TIR-NBS-LRR) proteins are subcategories of NBS-encoding proteins specifically targeting pathogen effector proteins inside the host cell for effector-triggered immunity. Five NBS containing proteins were upregulated at 2 and 3 dpi (Figure 5B,C). We also searched the SolariX (cibiv.at/SolariX) compendium of NBS domain-containing proteins and the Spud DB (Solanaceae.plantbiology.msu.edu) using gene IDs [55]. Table S6 shows seven NBS containing proteins, including the newly identified Soltu.DM.01G023270.1, and Soltu.DM.05G006210.1, which DRAGO2 and RGAugury identified as a TM-CC class protein although having an NBS domain [55,56]. Three NBS containing proteins are specifically involved in responses to *Pseudomonas syringae*, *P. infestans*, or *Meloidogyne javanica*, while two NBS proteins represent ABC-2 transporter proteins involved in metabolic functions [47,51,56].

The majority of upregulated RGAs belong to the RLP (TM-LRR) and RLK (TM-LRR-STTK) classes of proteins, suggesting that PVX infection stimulates PAMP/MAMP triggered immunity. Given that RLPs contribute to defense and plant development, we used the gene IDs to search within the SolariX or Spud DB for their functions (Table S6). We identified gene homologs for *Too many mouths* (TMM) and *ERECTA*, which engage in stomata development and distribution. One gene encodes a TM-CC protein that is a potential uridine kinase and normally attributed to developmental pathways. Homologs for *Ve1*, *EF-Tu*, *BAK1* are linked to PAMP/MAMP triggered immunity involving bacterial and fungal pathogens (Table S6).

**Figure 6:**
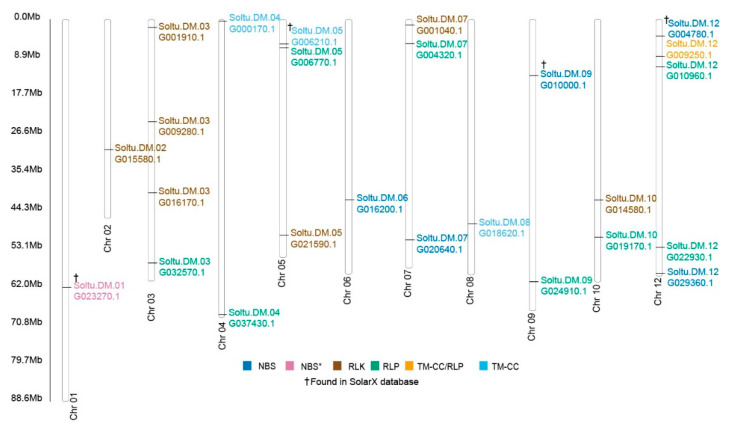
Distribution of the PVX-upregulated PRG analogs along *S. tuberosum* chromosomes. The scale indicates the genome size of *S. tuberosum* in Mb. Genes are color-coded according to the legend in the figure. “†” genes in the SolariX database search.

We mapped the chromosomal distribution of the RGAs analyzed onto the 12 chromosomes and found the loci did not cluster (Figure 6). Each Chr1, Chr2, Chr6, and Chr8 have one PRG locus, each Chr4, Chr9, Chr10 have 2 PRG loci, and each Chr5, Chr7 have three PRG loci. Chr3 and Chr 12 have four and five loci, respectively.

### 2.5. DEGs Associated with the Unfolded Protein Response (UPR) that Are Common between Potato and Arabidopsis

**Figure 7:**
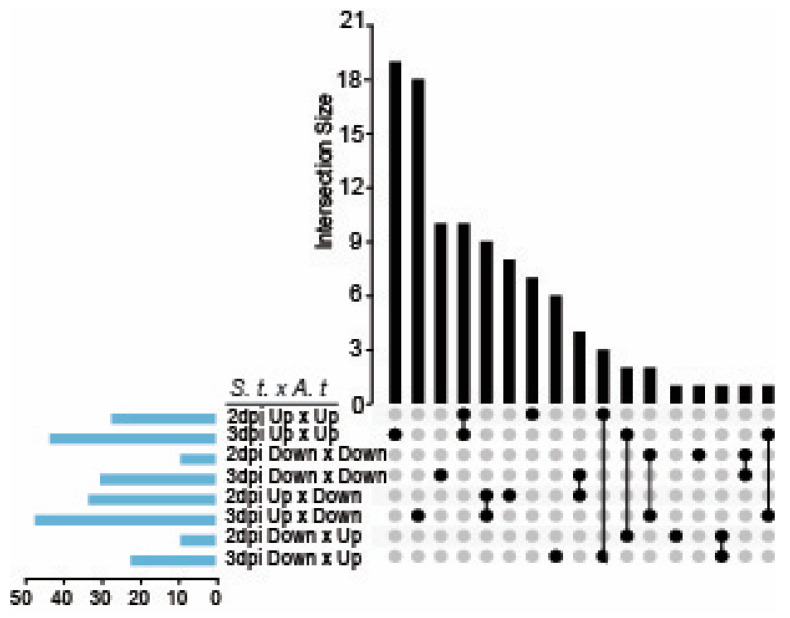
Functional characterization of UPR related genes in S. tuberosum DEGs. The UpSet plot shows potato DEGs (2 dpi and 3 dpi) that were also represented in a published Arabidopsis UPR DEGs dataset obtained following induction by tunicamycin (5 µg/mL) [58]. *S.t. x A.t* is shorthand for *S. tuberosum* by Arabidopsis comparison indicating DEGs that are upregulated or downregulated in both datasets or alternatively up and down regulated in each dataset.

The unfolded protein response (UPR) monitors and responds to disruptions in the protein folding capacity in the ER caused by environmental stress. Previously we showed that the UPR serves to restrict the accumulation of PVX and a related virus *Plantago asiatica mosaic virus* (PLAMV) in *N. benthamiana*, *A. thaliana*, and *S. tuberosum* [15,17,57]. To discover whether UPR downstream genes were among the DEGs in the PVX-induced transcriptome, we compared the potato transcriptome dataset with a published transcriptome dataset of ER stress-regulated genes reported by Song et al. (2015) produced in Arabidopsis treated with tunicamycin (5 µg/mL for 4 h) [58]. This Arabidopsis study identified 286 upregulated (≥2-fold) and 170 downregulated genes (≤0.5-fold) using Agilent oligo microarray technology. We compared the PVX-induced transcriptome at 2 and 3 dpi with the single time point of the Arabidopsis dataset to determine which DEGs might be affiliated with UPR signaling pathways and presented the data using an UpSet plot (Figure 7). As a shorthand for representing the comparisons of *S. tuberosum* and Arabidopsis dataset we used S.t. x A. t. indicating DEGs that are upregulated (Up x Up) or downregulated (Down x Down)) in both datasets or alternatively upregulated and downregulated (Up x Down, Down x Up) in each dataset. We separately compared the S. tuberosum DEG datasets at 2 dpi and 3 dpi with the same Arabidopsis DEG dataset.

At 2 dpi, 20 total genes were induced in the *S. tuberosum* with identified homologs in the Arabidopsis datasets (Table S7). Only seven genes were upregulated at 2 dpi which is identified in the graphic as “2 dpi UpxUp” and marked with a single circle under the plot. Ten genes were upregulated at 2 and 3 dpi which is indicated by linked circles between 2dpi UpxUp and 3dpi UpxUp (Figure 7). Three genes were upregulated at 2 dpi but downregulated at 3 dpi as indicated by the linked circles below the bar graph as 2 dpi UpxUp and 3 DownxDown (Figure 7). At 3 dpi there were a total of 31 upregulated genes. This total is the sum of 19 genes that were upregulated in both S. tuberosum and Arabidopsis (UpxUp) at 3 dpi only, ten genes that were upregulated in both hosts at 2 and 3 dpi, and two genes were upregulated at 3- dpi but downregulated at 2 dpi (represented by linked circles). Notably considering genes that are primarily downregulated in the Arabidopsis dataset, we identified eight upregulated genes at 2 dpi, 18 upregulated genes at 3 dpi, and 9 upregulated genes at 2 and 3 dpi. We identified six downregulated genes in the potato transcriptome at 2 dpi. Twenty-five genes were downregulated genes at 3 dpi (Figure 7, Table S8).

Next, we closely examined the gene model descriptions attributed to the Arabidopsis genes to functionally characterize the genes identified as common to the *S. tuberosum* and Arabidopsis datasets (Tables S7 and S8). Considering the upregulated genes across 2 and 3 dpi, we identified six major activities: (1) UPR and ER-associated protein degradation (ERAD), (2) plasmodesmata and cell wall functions, (3) cargo transport, (4) disease resistance, systemic acquired resistance, oxidative stress, (5) transcription and DNA functions, (6) abiotic stress responses. The UPR is a mechanism that refolds malformed proteins in the ER. The ERAD machinery degrades proteins that cannot be refolded. There are four factors involved in these responses that were commonly activated by PVX infection and ER stress treatment in Arabidopsis. Glycosyltransferase plays a role in protein glycosylation and maturation [59]. Cytochrome P450 has multiple specificities and is a co-factor binding Bax inhibitor 1 in the ER which is a regulator of UPR signaling [60]. HRD1 is a ubiquitin ligase and F-box protein complex with ubiquitin ligases to modify protein substrates for degradation [61].

Regarding plasmodesmata and cell wall functions, pectin lyases, pectin esterases, and remorins have been identified as factors that recognize invading viral pathogens and regulate virus cell-to-cell movement. The PVX 25K protein is reported to bind remorin [62,63,64]. WAK-like receptor-like kinases function in gene-for-gene resistance and recognize apoplastic efforts to confer pathogen resistance [65]. The carbohydrate-binding X8 domain protein binds to callose at the aperture of plasmodesmata. The X8 domain proteins regulate the expansion or constriction of the plasmodesmal aperture to control cell-to-cell communication and virus intercellular movement [66]. Regarding cargo transport, the ER-localized p24 protein is a type-I membrane protein that associates with the Golgi and cargo vesicles and is sometimes involved in transporting GPI-anchored proteins to the plasma membrane [67,68,69].

Regarding disease resistance, several genes were identified that play various roles in broad pathogen resistance including R gene-mediated resistance, oxidative stress, and systemic acquired resistance: *HIN1/NDR1*, *lipase*, *NAC28*, *UDP-glycosyltransferase*, *cysteine-rich RLK*, and *cytochrome p450* [24,45,47,60,70,71,72]. None of these factors were specifically antiviral except for *AtNAC28*, which restricts TCV infection. Genes that play known roles in drought, heat, and salt stress are *cytochrome p450*, *CYP709B3* (a relative of cytochrome p450), *UDP-glycosyltransferase*, and *LEA family* members [24,73,74,75]. Four genes encode known transcription factors: ZPT2, MADS-box TF, ZFHD1, and AtNAC28, and one factor likely regulates DNA functions, Ring/UBox E3 ligase. These data suggest that PVX may use the UPR machinery to reprogram gene expression during infection.

## 3. Discussion

This study examines the potato gene expression profile during the early days of PVX-GFP infection. We mined a transcriptome dataset for DEGs that are up- or down-regulated to understand the host genetic responses and identify key regulatory networks that respond to PVX infection. Surprisingly, the upregulated and downregulated genes changed significantly between 2 and 3 dpi. To better understand these genetic responses, we used GO analysis to cluster DEGS into common biological processes, cellular components, and molecular functions at 2 and 3 dpi. A significant portion of DEGs is generally reported to respond to environmental stimuli and stress, oxidative-reductive processes, transcription factors, protein modifications, and metabolic processes, and membrane transport. The pattern of GO terms led us to investigate the TFs, RGA, and UPR responsive genes that may be induced or suppressed to test the hypothesis that PVX infection stimulates early genes involved in broad cellular adaptive and defense responses.

Transcriptional regulatory networks are crucial for plant adaptation to adverse environments including heat, drought, cold, and pathogen attack [76]. Plant viruses are known to alter their cellular environments to create conditions that support infection, or to create leaf environment conditions that attract herbivorous insects which may also act as vectors for virus dispersal. PVX infection associated transcriptional regulatory networks have been largely unexplored. In this study, a total of 107 TFs were upregulated at 2 and 3- dpi while only 27 TFs are downregulated. For example, thirty-one upregulated factors belong to the AP2 family that primarily engage in regulatory networks associated with drought tolerance, ethylene response, and oxidative signals [77]. The AP2 factors typically ensure plant resiliency in adverse environments and it is worth speculating that these factors coordinate metabolic adjustments to ensure plant survival to PVX infection. The bZIP, HSF, MYB, NAC, WRYK factors identified in Table S4 belong to TF families that are known to bolster host survival. Previous investigations of PVX and the related potexvirus, *Plantago asiatica mosaic virus* (PlAMV), identified TFs such as bZIP60, bZIP28, and NAC089 which contribute to regulating antiviral responses in susceptible hosts [15]. These three TFs were not identified among the DEGs in the present study and prior investigations suggest that bZIP60 is activated in potato plants between 3 and 5 dpi. Five StbZIPs activated by PVX infection include StbZIP42, StbZIP58, StbZIP46, StbZIP61, and StbZIP37 [27]. Interestingly StbZIP42 and StbZIP61 are reported to be highly repressed following treatment with heat. StbZIP42 is also repressed following treatment with *P*. *infestans*, BABA or BTH [27]. StbZIP58 is highly induced following heat treatment [27].

Two previous studies reported responses to PVY infection in susceptible and resistant potatoes within the first 48 h of infection and while there were significant shifts in photosynthetic metabolism, redox-regulation, only individual WRKY, MYB, and DOF factors were identified as differentially expressed TFs [24,78]. Our study has uncovered a comparatively large number of TFs when compared with the previous studies looking at the plant virus interactions through transcriptome and proteome approaches [22,24,78,79,80,81]. The TFs identified in this Tables S4 and S5 have not been functionally characterized although there is sufficient sequence information to assign them to homology-based TF families. Further analysis is needed to determine the physiological implications of coordinated TF expression patterns, and their role in virus infection.

Innate immunity to plant viruses is controlled by single dominant or recessive R genes encoding factors that inhibit various stages in the virus infection cycle. Innate immunity is a form of basal immunity that is triggered by recognition of a specific effector protein that can derive from a specific virus species or strain. Effector triggered immunity (ETI) activates a broad range of local and systemic defense responses following pathogen recognition including protein kinase cascades [14,46,82]. Induced antiviral defense mechanisms such as systemic acquired resistance (SAR) involve the oxidative burst, expression of defense-related genes, and phytohormones such as salicylic acid (SA), jasmonic acid (JA), abscisic acid (ABA), azelaic acid (AZA), and glycerol-3-phosphate [46]. Prior studies showed that exogenous treatment of *N*. *benthamiana* leaves with SA activates alternative oxidase (AOX) in the mitochondria which play an important role in basal resistance to PVX [83]. In this study, we used GO term analysis to initially categorize DEGs and we identified clusters of sequences involved in cellular responses to stimuli or stress, kinases, and oxidative-reduction processes which are features usually associated with innate or induced immunity to virus infection. As the various defense-related phytohormones inducing a broad range of signaling pathways, the most straight forward approach to investigate whether basal immunity might be activated was to search the transcriptome for RGAs. Recent transcriptomic studies have led to the identification of RGAs in the model and non-model systems that are induced by pathogen effectors and virulence factors [51,52,53,54,83]. We searched for RGAs in the PRGdb and SolariX database and identified seven NBS containing, four RLP and two RLK genes. It is remarkable to see such early transcriptional changes that reflect R gene recognition of virulence factors although we do not know which viral features stimulate this host-mediated response. Generally, R gene-mediated responses involve either extreme resistance or hypersensitive cell death. However, recent transcriptome analysis in cassava challenged with South African cassava mosaic virus (SACMV) identified transcriptionally upregulated RGAs that play crucial roles in tolerance and recovery to geminivirus infection [84]. In this model, RGAs are proposed to provide surveillance in a manner that might lower SACMV replication and reduce symptoms. Such a model might also explain the mild symptoms caused by PVX infection in potato plants. On the other hand, evidence that multiple RGAs are stimulated that were identified as having putative roles in bacterial and fungal resistance suggesting that broad-spectrum resistance may be activated as a consequence of PVX infection. Perhaps PVX infection stimulates multiple RGAs to exclude competing pathogens that may challenge the same host [47,83]. Another possibility is that virus infection stimulates the expression of RGAs which are maintained by the cell in a signal competent but an auto-inhibited state. It is possible that early activation of transcription leads to higher levels of RGAs but these specific R proteins identified in this study failed to activate cellular defenses. Perhaps one or more of these RGA proteins heterodimerize with other R protein partners to activate antiviral defenses and that such partners are not available [85]. In this model, the RGAs detect but fail to counteract virus invasion. Further research is needed to explore the role of these RGAs in creating a state of PVX tolerance or broad-spectrum resistance.

The ER is a central hub for responses to adverse environmental challenges such as virus infection, heat, chemical, osmotic, and salt stress [86]. The physiological consequences of prolonged low-level ER stress include constrained plant development and productivity, whereas chronic stress can result in death and crop losses. The transmembrane sensors of ER stress in plants are the inositol requiring enzyme 1 (IRE1), bZIP28, and bZIP17 [87]. These well-known monitoring/sensing pathways use the transcription factors bZIP60, bZIP28, and bZIP17 to activate cellular adaptive responses. They coordinate the transcription of molecular chaperones, including the ER lumen binding protein (BiP). Current research has shown that the PVX TGB2 and TGB3 proteins are movement proteins that are embedded in the ER. TGB3 is specifically recognized by the IRE1/bZIP60 as well as the bZIP17 pathways which coordinate to restrict virus movement [15]. We downloaded a published Arabidopsis dataset that represents ER stress- induced genes that promote protein homeostasis in leaves treatment with tunicamycin [58]. By comparing the PVX induced transcriptome dataset in potato with the Arabidopsis dataset [58] we identified common candidate genes that contribute to ER stress response and virus infection. Surprisingly when we compared these datasets, we identified only 51 common DEGs, and none of these encode protein folding enzymes or chaperones that reside in the ER, which are typically UPR downstream genes. The UPR related genes in the dataset include glycosyl transferase and cytochrome p450. The presence of glycosyltransferase reflects a need to enhance protein modifications coupled with protein folding. Interestingly, HRD1 and F-box proteins that are attributed to ERAD were increased which are necessary for degrading malformed proteins that cannot be refolded in the ER. We identified several cargo functions that might complement increasing protein synthesis brought on by virus infection. There are also increases in plasmodesmata functions which are likely important for regulating virus cell-to-cell trafficking. We identified several factors that engage in cellular adaptation to abiotic and biotic stress as well as disease resistance. We also identified a set of transcription factors that have not been previously studied for their role in ER stress management. In Table S4 there are three heat shock transcription factors identified which have homologs in Arabidopsis that have been linked to UPR regulation in the cytoplasm. Activation of these HSF factors may precede activation of the ER-resident sensors. A recent report links HSF transcription factors to sensing oxidative stress and enhancing UPR gene expression [88]. These data suggest that early UPR regulation is linked to several cellular adaptive responses occurring outside the ER and these responses encompass physiological processes beyond protein homeostasis.

## 4. Materials and Methods

### 4.1. PVX Inoculation of Potato Leaves

*S. tuberosum* cultivar ‘Russet Norkota’ (USDA Gene Bank Accession # AV49) plants were propagated by cuttings on Murashige and Skoog medium (PhytoTech Labs, Lenexa, KS). Rooted cuttings were potted and grown in a growth room with a 12 h photoperiod at 20 °C for four weeks. The PVX-GFP infectious clone, maintained in *Agrobacterium tumefaciens* strain GV3101 [72], was delivered to *N. benthamiana* plants grown under 12 hr light at 20 °C. Two *N. benthamiana* leaves were dusted with carborundum and mechanically inoculated. Then, the upper leaves were harvested after the appearance of symptoms (2 weeks), ground 1:10 (*w*/*v*) in 0.01 M phosphate buffer (pH 7.0), centrifuged at 6000 rpm for 5 min, and then the soluble phase (sap extract) was stored at −80 °C. Standard infectivity assays [71] to estimate the amount of infectious virus in the sap preparation were conducted to ensure that future reproducibility in which other researchers can sample leaves having the same number of infection sites, which likely influences the measurable transcriptome responses. *Chenopodium quinoa* leaves (*n* = 6) were rub-inoculated with 20 µL of sap and the numbers of chlorotic foci were counted after 7–12 days to standardize the inoculum titer. Then, nine large terminal leaflets of compound potato leaves were mechanically inoculated with 20 µL of sap after dusting with carborundum and three leaves were pooled for RNA extraction to create three separate RNA samples for RNAseq. Mock treatment was carried out using only 0.01 M phosphate buffer (pH 7.0). Leaf samples were collected after 2–3 dpi, immediately frozen in liquid nitrogen, and stored at −80 °C freezer for further use [27]. Fluorescence imaging was carried out using Stemi SV11 Apo M^2^BIO and a 20× objective (Kramer Scientific Corp., alley Cottage, NY, USA) using GFP-470nm and GFP-500nm filters.

### 4.2. RNA Extraction, cDNA Library Construction, Filtering Sequencing Reads

Total RNA was extracted using the RNeasy Mini Kit (Qiagen). RNA purity was assessed using Epoch 2 Microplate Spectrophotometer (BioTek, VT, USA). All samples produced A_260_/A_280_ ratios ranging between 1.9–2.1. RNA integrity was assessed using Agilent 2100 bioanalyzer (Agilent Technologies, Palo Alto, California) and all samples had an RNA integrity number (RIN number) >7.3 as described in Herath and Verchot (2021) [27].

The mRNA purification, fragmentation, cDNA synthesis, second-strand synthesis, adapter ligation, cDNA library purification, and transcriptomic sequencing was performed at the Beijing Genomics Institute (BGI, Shenzhen, China) using the BGISEQ-500 platform [89]. BGI performed PE150 strand-specific library preparation, generated raw data, and provided clean reads as follows. First, the polyA–containing mRNA was purified using oligo(dT)-coupled magnetic beads. Then, mRNA fragmentation was carried out using divalent cations under elevated temperature. The cleaved fragments were converted into the first-strand cDNA using reverse transcriptase and random primers. Then, second-strand cDNA synthesis using DNA polymerase I and incorporating dUTP (2′-deoxyuridine 5′-triphosphate) in place of dTTP (2′-deoxyguanosine 5′-triphosphate). The cDNA library was prepared using DNA nano ball technology in the BGISEQ-500 platform, suited for high throughput transcriptome studies [27].

BGI used SOAPnuke (ver 2.1.0) for quality control and preprocessing of the raw data to eliminate adapters or low-quality sequences. The parameters for cutting adapters and setting length restriction were: 0.01 -l 20 -q 0.4 -A 0.25 --cutAdaptor -Q 2 -G --polyX50 --minLen 150, as explained in the SOAPnuke github.com repository [90]. FASTQC was used to assess read qualities (version 0.11.9). The subsequent analysis returned clean reads. The clean reads generated by BGISEQ-500 were submitted to the Sequence Read Archive database at NCBI under the bio project PRJNA679820 [27].

### 4.3. Genome Mapping and Gene Expression Analysis

Clean sequencing reads were mapped to the *S. tuberosum* reference genome (DM v6.1) available at SPUDdb (http://solanaceae.plantbiology.msu.edu—last access date 28 January 2021) [25] using Hierarchical Indexing for Spliced Alignment of Transcripts 2 (HISAT2) [91]. Bio-SAMtool (version 1.9) was used for formatting and storing alignments as SAM files and companion BAM files [92]. Transcripts assembly and abundance were determined using StringTie (version 2.1.4) and annotated according to the reference genome (DM v6.1) [93] (Table S1). The bash script used for the analysis is available at https://github.com/venuraherath/PVX_Transcriptome_Analysis—last access date 2 February 2021. Then, the results were converted to DESEQ2 format using prepDE.py python scripts available in the program for differential expression analysis.

Differential sequence expression analysis was carried out using DESeq2 (version 1.28.1) in RStudio (version 1.3.959). DESeq2 performs differential analysis of count data. A list of differentially expressed genes at 2 and 3 dpi was generated by testing the log_2_-fold changes between treatment and control and ranked as less than −1.2 or greater than 1.2 with a resulting *p*-value of ≤0.05 that was adjusted using Benjamini and Hochberg’s (FDR) method [94,95]. We used TBTools ver 1.0692 to prepare Volcano plots, Venn diagrams, and heat maps [96].

### 4.4. Gene Ontology (GO)Enrichment Analysis

BLASTp was used to find potential homologs using the *e*-value cut-off of 1 × 10^−3^ against Viridiplanta proteins in NCBI non-redundant (nr) database. OmicsBOX ver. 1.4.11 (BioBam BioInformatics Solutions; biobam.com/omicsbox/—last access date 15 January 2021) was used to perform GO annotation. The fasta formatted protein sequences were used for a BLAST search against the NCBI nr database and the InterPro database (https://www.ebi.ac.uk/interpro/—last access date 15 January 2021) representing protein domains and families [97] using and an *e*-value hit filter of 1 × 10^−6^ and cut-off of 55. The search results were merged, and sequence annotations were carried out using GO terms, considering GO hierarchy, quality, and abundance of the source annotations. To obtain functional information, the GO terms were classified at GO level 5 and a node-score of 5 for the complete list of DEGs Then, GO term enrichment analysis was carried out based on Fisher’s exact test, and a *p*-value cutoff of 0.05.

### 4.5. Functional Annotation of Transcription Factors and Resistance Gene Analogs (RGAs)

Differentially regulated transcription factors among the DEGs and their orthologs in Arabidopsis were identified using The Protein Annotation with Z-scoRE server (PANNZER2, http://ekhidna2.biocenter.helsinki.fi/sanspanz/—last access date 10 January 2021) [98]. RGA identification was carried out using DRAGO 2 pipeline with default settings against both reference and putative genes included in the PRGdb 3.0 server (http://prgdb.org/prgdb/—last access date 20 January 2021) [55].

### 4.6. Comparative Analysis of PVX Induced DEGs in Potato and Published Tunicamycin Induced DEGs in Arabidopsis

DEGs induced by treatment of Arabidopsis seedlings with 5 ug/mL tunicamycin for 4 h were retrieved from Song et al.’s 2015 study (Supplementary Information (http://www.pnas.org/lookup/suppl/doi:10.1073/pnas.1419703112/-/DCSupplemental—last access date 10 December 2020) [59]. Reciprocal Blast was conducted against the Arabidopsis UPR induced genes to identify potential homologs genes with a minimum identity percentage of 90 % using NCBI blast ver 2.9.0^+^. To visualize the intersection between the PVX-induced DEGs in potato at 2 and 3 dpi with the tunicamycin induced DEGs in Arabidopsis and the sequences categorized as up or down-regulated, an UpSet plot was generated using TBtools version 1.074. Then, functional categorization of the identified genes was carried out using the GO annotation tool (https://www.arabidopsis.org/tools/bulk/go/index.jsp—last access date 12 December 2020) available at The Arabidopsis Information Resource (TAIR) database (https://www.arabidopsis.org/—last access date 12 December 2020) [99]. Microsoft Excel and Adobe Illustrator 2020 were used to prepare and compile charts.

## 5. Conclusions

PVX is one of the major plant viruses that infect the Solanaceae family including potatoes. In this study, we showcase the transcriptional regulatory networks triggered during the early stages of the PVX infection. We identified 1242 DEGs spanning across 2 and 3 dpi. Interestingly, the majority of up-regulated DEGs are involved in stress response, redox regulation, as well as inter-and intracellular transport processes that coincide with the entry and the establishment of PVX infection. We also identified the key TFs and target genes involved in the pathogen response and UPR pathways. Further studies are required to functionally characterize the identified major transcription factors that potentially playing a major role in regulating general and specific responses to PVX infection.

## Data Availability

RNA-Seq data were deposited in Sequence Read Archive database at NCBI under the bio project PRJNA679820 [27].

## Supplementary Materials

The following are available online at https://www.mdpi.com/1422-0067/22/6/2837/s1, Figure S1: Standardization of PVX-GFP inoculum. (**A**,**B**) Infection foci on Chenopodium leaves at 4 and 7 dpi were counted to determine the infectivity of the standard 20 µL inoculum dose. (**C**,**D**) Representative potato plants that were inoculated with PVX-GFP and mock inoculum. (**E**,**F**) Fluorescence microscopic images showing PVX-GFP containing infection foci on inoculated potato leaves, Table S1: Summary of the RNA-sequencing statistics, Table S2: *S. tuberosum* genes that are upregulated at 2 and 3 days post-inoculation (dpi), Table S3: *S. tuberosum* genes that are downregulated at 2 and 3 days post inoculation (dpi), Table S4: Upregulated transcription factors at 2 or 3 dpi, Table S5: Downregulated transcription factors at 2 and 3 dpi, Table S6: Putative RGAs according to Spud and SolariX databases, Table S7: Primarily upregulated genes in PVX-infected *S. tuberosum* and tunicamycin treated Arabidopsis, Table S8: Primarily downregulated genes identified in PVX infected *S. tuberosum* and in tunicamycin treated Arabidopsis.
