# Supplementary material for: Transcriptional Regulatory Networks Associate with Early Stages of Potato Virus X Infection of Solanum tuberosum
Source: Int J Mol Sci. 2021 Mar 11;22(6):2837. doi: 10.3390/ijms22062837 (PMC8001266; doi:10.3390/ijms22062837)
Supplement: Supplementary file 1 [file ijms-22-02837-s001.zip › Supplementary Figure S1.pdf]

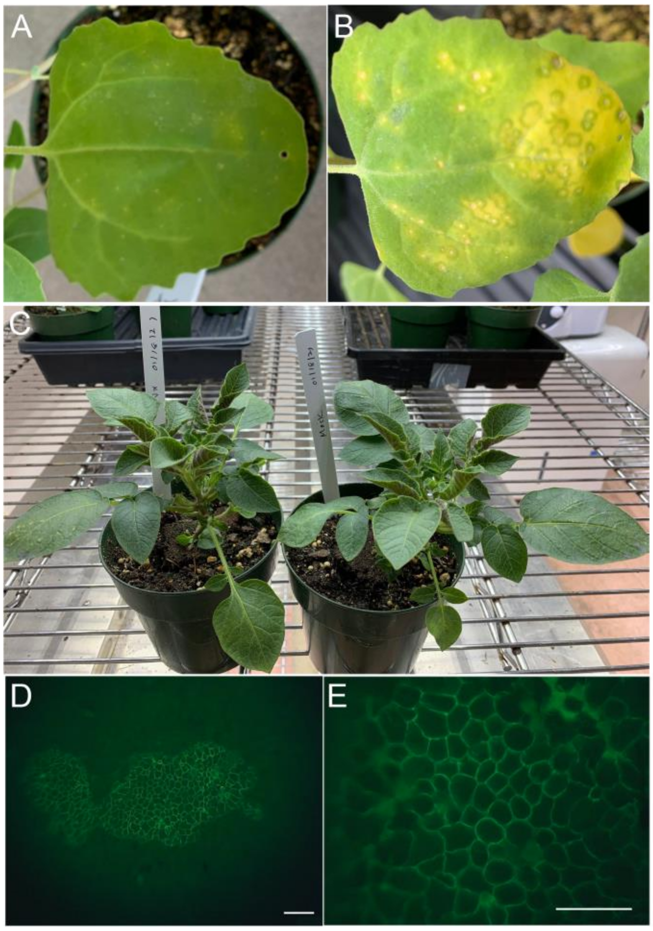

Figure S1. Standardization of PVX-GFP inoculum. (**A**), (**B**) Infection foci on Chenopodium leaves at 4 and 7 dpi were counted to determine the infectivity of the standard 20 µl inoculum dose. (**C**), (**D**) Representative potato plants that were inoculated with PVX-GFP and mock inoculum. (**E**), (**F**) Fluorescence microscopic images showing PVX-GFP containing infection foci on inoculated potato leaves.
